# “Same Calories, Different Foods” – What do you choose? The role of construal level and age in shaping food choices

**DOI:** 10.3389/fpsyg.2025.1688277

**Published:** 2025-10-29

**Authors:** Eunho Kim, Yoon-Hee Kang

**Affiliations:** ^1^Department of Management Information Systems, Dong-A University, Busan, Republic of Korea; ^2^Department of Psychology, Chung-Ang University, Seoul, Republic of Korea

**Keywords:** food choice, food type, food quantity, construal level theory, age differences, psychological mechanisms, health behavior, health communication

## Abstract

**Introduction:**

Health-related food choices are often influenced by two central attributes: food type (such as healthy vs. less healthy) and food quantity (such as large vs. small portions). Based on Construal Level Theory (CLT), this research investigates how both chronic and situational construal levels guide consumers’ attention toward these attributes and how this process varies by age. By examining decisions under caloric equivalence, the study aims to explain when and why equally caloric foods are chosen differently among consumers of different age groups.

**Methods:**

Four experiments were conducted across two main studies. Study 1A examined how individuals’ chronic construal levels, measured by the Behavioral Identification Form (BIF), influence food choices between healthy large portions and less healthy small portions of the same calories. Study 1B experimentally manipulated situational construal levels through temporal distance and tested their causal effects on food choices. Study 2A explored whether age predicts individuals’ tendency to adopt highor low-level construals. Study 2B further investigated whether attentional focus on food type versus quantity mediates the relationship between age and food choice using a nationally representative sample.

**Results:**

Across Studies 1A and 1B, individuals with a low-level construal were relatively more likely to focus on food quantity and select smaller portions even when these were less healthy, whereas those with a high-level construal focused on food type and preferred healthier options. Study 2A found that older adults were more likely to exhibit lower-level, concrete thinking compared to younger adults. Study 2B showed that age-related differences increased older adults’ attentional focus on food quantity, which in turn led them to choose smaller but less healthy foods. The findings collectively reveal that age affects food decisions indirectly through differences in attentional focus and construal level.

**Discussion:**

Together, these studies integrate construal level and age within a unified framework of health-related food decision-making. The results demonstrate that construal level serves as a key psychological mechanism explaining why people of different ages make different food choices even when calorie levels are identical. This research provides valuable insights for developing age-tailored health communication strategies and designing more effective dietary interventions that account for cognitive and motivational differences across age groups.

## 1 Introduction

“Should I consume a small portion of high-calorie food, or a larger portion of low-calorie food?” In contemporary society, food is no longer perceived solely as a means of sustenance; rather, it plays a central role in the pursuit of a healthy and sustainable lifestyle. Amid growing global awareness of wellness and preventive health, consumer attention to dietary choices has significantly increased. The rising prevalence of chronic diseases and obesity across regions—including North America, Europe, and Asia—has transformed healthy eating from an individual concern into a pressing public health issue (World Health Organization, 2020). According to Nielsen’s Global Health and Wellness Report (Nielsen, 2015), over 70% of surveyed adults reported modifying their diets to improve their health. Specifically, 65% indicated efforts to reduce fat intake, while 57% reported an increased consumption of fresh and natural foods. Complementary findings from other regions support this trend. For instance, Verbeke (2005) highlighted health as a primary determinant in food purchasing decisions among Belgian consumers, and Nguyen et al. (2019) found that Vietnamese consumers actively seek food safety and health-related information when purchasing food. Together, these studies underscore the global shift toward health-conscious food choices and the growing salience of health in consumer decision-making. This trend is mirrored by the food industry’s expanding use of health-related labeling and the proliferation of functional food products.

When choosing food, consumers typically consider two key attributes: food “type” and food “quantity”. The categorization of foods as either “healthy” (e.g., salads, whole grains) or “unhealthy” (e.g., fried foods, high-fat processed products) reflects a widely shared cognitive schema (Haws and Liu, 2016a). For example, research on fast-food consumers in the United States demonstrates that perceptions of food type strongly influence food selection. As such, food “type” operates as a categorical cue that informs consumer evaluations. In contrast, food “quantity”—such as portion size—also plays a critical role, as it shapes perceptions of calorie intake and satiety (Chernev and Gal, 2010; Van Phuong et al., 2025). Empirical evidence suggests that both attributes influence food choice. For instance, Van Phuong et al. (2025) found that both type and quantity were significant considerations for Vietnamese consumers, while Duarte and Teixeira (2021) reported that health-related labeling and nutritional information strongly affected purchasing intentions. These findings highlight that categorical (type) and quantitative (amount) information function as distinct yet equally salient factors in consumer decision-making, particularly in health-relevant contexts.

The dual focus on type and quantity in food choice can be interpreted through the lens of Construal Level Theory (CLT; Trope and Liberman, 2010). A focus on food “type”—such as evaluating whether a food is healthy or unhealthy—entails abstract, category-based processing, reflecting a high-level construal. In contrast, attending to food “quantity”—such as assessing how much to eat—requires concrete, detail-oriented thinking, indicative of a low-level construal. Thus, even when calorie content is held constant, consumer preferences may diverge depending on whether they adopt a high- or low-level construal, ultimately influencing their food choices.

Moreover, construal levels are known to vary systematically with age. Prior studies suggest that older adults, often guided by cognitive constraints and experiential learning, tend to adopt more concrete, action-focused processing styles—consistent with low-level construals. In contrast, younger adults are more likely to rely on abstract, goal-oriented thinking aligned with high-level construals (Hadar et al., 2021). Supporting this view, Mikels et al. (2016) and Liu et al. (2021) found that older consumers place greater trust in concrete dietary guidelines (e.g., “how much to eat”), whereas younger consumers are more attuned to abstract nutritional concepts (e.g., “balanced diet”). These findings imply that age-related differences in construal level may significantly affect how individuals interpret health information and, consequently, which attributes they prioritize in food selection.

Building on these theoretical and empirical insights, the present study aims to examine how consumers’ construal levels shape their emphasis on food “type” versus “quantity” in making dietary choices. More specifically, we investigate whether these preferences differ across age groups, in line with age-related variations in construal level. By exploring the interaction between construal level and age, this research seeks to uncover how different consumer segments respond to food-related information and what factors drive their choices when caloric content is held constant. Unlike previous studies that have examined either food type or portion size in isolation, the present research aims to integrate both attributes under caloric equivalence within a unified CLT framework. We conceptualize construal level as the key psychological mechanism that determines which attribute becomes focal and intend to test its influence through both chronic (BIF) and situational (temporal-distance) manipulations. In addition, we aim to explore how age-related differences in construal level shape consumers’ focus on food attributes, expecting that older adults may attend more to quantity while younger adults focus more on type. Through this integrative approach, the study seeks to clarify when, why, and for whom equally caloric foods are evaluated differently, ultimately providing theoretical and practical insights for designing age-tailored health communication strategies.

## 2 Literature review and hypothesis development

### 2.1 Health management and food choice

As life expectancy increases, the focus has shifted from merely living longer to living healthier. Aligned with the rise of wellness and well-being trends, consumers have become more attentive to the health implications of their diets and have actively sought out food choices that support health. In this context, dietary behavior is now widely recognized as a critical component of personal health management. Reflecting this shift, the health-related food industry has grown substantially, and academic interest in health-oriented dietary lifestyles continues to increase (Choi and Lee, 2024; Choi and Jin, 2010). Recent studies have also emphasized dietary change as a key behavioral strategy in health promotion (Goodyear et al., 2019).

Healthy eating refers to the practice of selecting foods that contribute to physical well-being, whether through home-cooked meals or dining out. As such, dietary behavior extends beyond personal taste or preference; it is increasingly framed as a proactive means of maintaining and promoting health.

Given the strong connection between dietary behavior and health goals, food choices are often analyzed through the lens of “virtue versus vice” (Haws and Liu, 2016b). Wertenbroch (1998) introduced this distinction to describe consumption choices in terms of temporal trade-offs. Virtuous products provide greater long-term benefits than immediate gratification, while vice products offer short-term pleasure at the cost of long-term value. Vice foods, in particular, are typically classified as temptation goods due to their sensory appeal, whereas virtue foods are associated with utility and long-term health benefits.

Consumers tend to rely on sensory cues, such as taste, when evaluating vice foods. However, those who are more health-conscious typically base their food choices on health-related attributes rather than hedonic ones (Mai and Hoffmann, 2015). Although such consumers may initially feel drawn to vice foods, they simultaneously experience aversion due to anticipated negative health outcomes—creating a motivational conflict (Metcalfe and Mischel, 1999). As a result, they are more likely to forgo vice foods in favor of virtuous options that align with long-term health goals.

Kim and Choi (2016) demonstrated that, under a health-focused goal, reducing the perceived “vice” of a food was more effective in promoting healthy choice than emphasizing its virtuous qualities. These findings suggest that consumers’ food choices are strongly influenced by their perception of the product’s alignment with health goals, especially when managing their own well-being. Beyond these individual-level factors, recent research has shown that age and situational contexts jointly shape healthy eating behaviors. Mal and Sen (2024) provided qualitative evidence that psychological and socio-cultural situations—such as emotional state, time of day, and social setting—systematically interact with age to influence dietary patterns. Younger adults exhibit greater variability in eating behavior depending on peer influence, stress, or convenience, whereas older adults maintain more stable and health-oriented eating routines guided by medical advice, social norms, and physiological needs. These findings suggest that age-related differences in eating cannot be understood in isolation from situational contexts, which jointly determine how individuals of different ages manage their health through food. Accordingly, understanding the interplay between age and situation provides a richer foundation for explaining why people of different ages adopt distinct strategies for healthy eating.

### 2.2 Food choice based on type and quantity: a construal level perspective

When making inferences about the overall healthiness of food products, consumers rely on various attribute cues. Previous research has primarily focused on the “type” of food—classifying options as either healthy (e.g., salad) or unhealthy (e.g., French fries)—and encouraging choice based on this binary “virtue–vice” distinction. In this view, food type has been positioned as the core determinant in consumers’ perceptions of healthiness and their decision-making. However, more recent studies have begun to emphasize the role of “quantity” (e.g., portion size) as another critical factor influencing food evaluation and choice (Chernev and Gal, 2010; Liu et al., 2015). These two attributes—type and quantity—are processed independently by consumers and may serve different psychological functions (Woolley and Liu, 2021).

Health-conscious consumers frequently estimate the calorie content of various meal options when deciding what to eat. For example, when choosing between a small McDonald’s cheeseburger and a larger 12-inch Subway turkey sandwich, consumers might estimate the calories in both and opt for the one perceived to be lower in calories. These estimations are primarily influenced by two key features: the food’s type and quantity (Woolley and Liu, 2021).

Specifically, food type refers to a categorical judgment about what the food is—typically evaluated in terms of whether it is healthy or unhealthy. In contrast, food quantity refers to a numerical judgment about how much food is present—such as the portion size or volume. These two attributes are processed differently.

Previous studies suggest that food type is processed more quickly and automatically than quantity and often serves as the initial anchor for judgment (Liu et al., 2015). In contrast, food quantity tends to be processed more slowly and secondarily (Liu et al., 2015). Consumers typically engage in a stepwise processing model in which they first assess the healthiness of a food and subsequently consider quantitative attributes like calorie content (Liu et al., 2015; Oakes, 2005; Ordabayeva and Chandon, 2016). This type-based processing approach is not unique to food but also appears in other domains such as exercise. For instance, when evaluating an exercise routine, individuals often initially categorize it as either “easy” or “intense” before considering more detailed attributes like duration or exertion level.

However, the final food decision may differ depending on which attribute—type or quantity—is more salient in the consumer’s mind. Although both can influence calorie estimations and food choices, the salience of either attribute can shift depending on the consumer’s goals, motivational state, and contextual factors. For example, in a state of extreme hunger, quantity may become the focal attribute, whereas for a highly health-conscious consumer, type may be more salient. Thus, type and quantity may vary in their influence depending on the situation and cognitive priorities.

This study proposes that the attribute consumers focus on most—and consequently their food choices—will differ depending on their level of construal. According to Construal Level Theory (CLT), people’s judgments and decisions vary based on whether they are operating under a high-level or low-level construal (Liberman and Trope, 1998; Trope and Liberman, 2010). High-level construals involve abstract thinking, focused on core values and overarching goals. Consumers at this level evaluate food based on its essential characteristics and broader implications, such as long-term health benefits. In contrast, low-level construals involve concrete thinking, emphasizing peripheral or situational features. These consumers focus on tangible aspects such as portion size and immediate outcomes.

The relationship between construal level and food choice has been well established in prior research. The level of construal influences consumers’ intentions to purchase healthy food. Zheng et al. (2025), grounded in Construal Level Theory (CLT), examined the interactive effect of temporal distance (present vs. future) and message framing (positive vs. negative) on consumers’ healthy food purchase intentions. The findings revealed that under negatively framed messages, a present temporal distance significantly enhanced intentions to purchase healthy food, and this effect was mediated by conceptual fluency. Furthermore, the interaction effect was significant only among consumers with low general health interest (GHI), whereas those with high GHI were unaffected by message design. These results indicate that the combination of temporal cues and framing strategies alters consumers’ information-processing mechanisms, thereby enhancing persuasive effectiveness. Consequently, the study underscores the importance of employing present-oriented negative message framing in the design of health-related advertising to effectively promote healthy food consumption.

Li et al. (2025) proposed that the level of construal influences how consumers weigh different attributes of food choices depending on social distance. Drawing on Construal Level Theory (CLT), the study posits that when social distance is low, individuals engage in low-level construal, focusing on concrete and immediate considerations such as taste, whereas high social distance induces high-level construal, emphasizing abstract and long-term goals such as health. Accordingly, individuals tend to prioritize taste when making food choices for themselves but emphasize health when making choices for others. Across a series of experiments, the study demonstrated that as social distance increased, the importance of health attributes rose, while taste attributes became more salient under conditions of close social distance. This effect was mediated by differences in construal level and further moderated by factors such as the target person’s characteristics and the closeness of the interpersonal relationship. The findings provide compelling empirical evidence that food choice behavior is not merely driven by individual preference but rather by the interaction between cognitive distance and psychological abstraction, offering a nuanced understanding of how social context and construal processes jointly shape health-related decision-making.

According to Perfetti et al. (2025), the level of construal plays a pivotal role in consumers’ choices between virtue (health-oriented) and vice (less healthy) products in vending machine contexts. Drawing on Construal Level Theory (CLT), the authors analyzed how consumers’ cognitive evaluations vary depending on the physical arrangement of products and the social context of the vending environment. CLT posits that individuals engage in low-level construal, characterized by concrete and immediate thinking, when psychological distance is low, whereas high-level construal involves abstract and future-oriented thinking when psychological distance is greater. Consequently, vice products are associated with low-level construals focused on immediate gratification, while virtue products correspond to high-level construals reflecting long-term health goals. An analysis of 13,709 actual vending machine purchases using a machine learning model revealed that products placed in the upper or right sections of the vending machine were more likely to be chosen when they were health-oriented, supporting the theoretical predictions of CLT. Furthermore, consumers in hospitals and universities tended to select more vice products, likely due to stress, whereas workplace environments encouraged greater virtue selections, reflecting stronger self-regulatory norms. These findings suggest that consumers’ impulsive purchases are shaped not merely by personal preference but by the interaction between psychological construal and environmental context, highlighting the potential for vending machine design and product placement strategies to promote healthier choices.

Collectively, these studies highlight that consumers’ food-related judgments are systematically shaped by their level of construal and contextual cues such as temporal, social, and environmental distance. Building on this stream of research, the present study extends the application of Construal Level Theory by examining how consumers’ focus on different food attributes—specifically, type versus quantity—varies as a function of construal level. Judgments based on food type are generally abstract and categorical—e.g., whether a food is healthy or unhealthy—and align with a high-level construal focused on purpose and values. For instance, when a consumer categorizes a food as “healthy,” this reflects an abstract judgment based on the food’s inherent characteristics and its alignment with long-term health goals. In contrast, judgments centered on food quantity involve concrete and actionable evaluations—e.g., “Is this amount enough for me?” or “How much will I eat?” These judgments are rooted in practical, immediate considerations and correspond to low-level construals. Therefore, differences in food judgments based on type and quantity are deeply tied to consumers’ psychological information processing strategies. Depending on their construal level, consumers may focus more on either food type or quantity, leading to divergent downstream decisions.

Understanding this interaction has important implications for promoting healthy behavior and designing targeted public health messages. For example, campaigns encouraging healthier food choices may benefit from tailoring their message framing—emphasizing either food type or quantity—based on the target audience’s dominant construal level. Accordingly, we hypothesize the following:

*H1*: Food choice will differ depending on construal level: Consumers with a low-level construal will be more likely than those with a high-level construal to choose less healthy food alternatives when the portion size is small.

### 2.3 Age-related differences in construal level

Construal Level Theory (CLT), as articulated by Trope and Liberman (2003, 2010), posits that the psychological distance perceived by individuals toward an object or event modulates the level of mental construal. Specifically, when psychological distance is low, individuals tend to engage in low-level construal, characterized by concrete, context-specific, and action-oriented processing. Conversely, greater psychological distance elicits high-level construal, which involves abstract, decontextualized, and goal-driven cognition. Psychological distance is conceptualized along four dimensions: temporal, spatial, social, and hypothetical, each influencing cognitive processing and behavioral outcomes across various domains such as health management, consumer behavior, and financial decision-making (Trope and Liberman, 2010).

Within the domain of food choice, consumers’ construal levels may differ depending on the focal attributes of the food and are likely moderated by factors including age, cognitive capacity, and motivational priorities. Recent research underscores age as a critical determinant, suggesting that older adults exhibit a propensity toward low-level construal in information processing (Steers, 2014; van Schie et al., 2015; Hadar et al., 2021). This tendency is closely linked to age-associated cognitive and motivational changes.

Three key factors underpin older adults’ inclination toward low-level construal. First, experiential information processing is predominant among older adults. According to previous studies, older adults—relative to their younger counterparts—rely more heavily on concrete, experience-based information when engaging in memory and judgment tasks (Peters and Daum, 2008; Huang et al., 2012). This reliance reflects a preference for practical and immediate data over abstract or idealized concepts. Second, declines in cognitive resources and shifts in motivational priorities influence information processing styles in older populations. Park (2002) found that older adults allocate greater attention to simple, heuristic cues rather than complex information when faced with cognitively demanding tasks. Additionally, Mather and Carstensen (2005) introduced the “positivity effect,” whereby older adults preferentially attend to positive over negative or complex stimuli. This motivational bias promotes selective processing of emotionally positive and concise information, reinforcing low-level construal tendencies. Third, a restricted future time perspective affects cognitive processing and decision-making in older adults. Steers (2014) provided experimental evidence that older individuals perceive future goals as temporally closer and more concrete, favoring short-term actionable objectives over long-term idealistic ones, particularly in health-related contexts.

van Schie et al. (2015) further explored this phenomenon through an experimental manipulation of construal level in older adults, presenting either abstract health goals or concrete action plans. The older cohort exhibited a “reversal effect,” maintaining a preference for concrete, feasibility-based evaluations even when primed with abstract information, indicative of a predominantly pragmatic decision-making style anchored in low-level construal. Complementing these findings, Hadar et al. (2021) examined attribute evaluation among age groups, noting that older adults demonstrated heightened sensitivity to peripheral, non-core product features, a pattern attributed to diminished inhibitory control and cognitive flexibility. This over-attention to ancillary details may prolong engagement with low-level construal processing and complicate decision-making.

Collectively, these findings elucidate the impact of age-related construal differences on health-related decision-making, including food choices. Older adults’ low-level construal orientation suggests greater sensitivity to quantitative aspects (e.g., portion size) rather than qualitative attributes (e.g., healthiness), potentially leading to preferences for smaller portions of less healthy foods. Conversely, younger adults’ higher-level construal favors abstract health goals, emphasizing food type over quantity, thereby increasing the likelihood of selecting larger portions of healthy options. In summary, CLT provides a robust theoretical framework for understanding age-related variation in cognitive processing and decision behavior. Accordingly, we posit the following hypotheses:

*H2*: Increasing age is associated with a greater propensity toward low-level construal.

*H3*: Older adults are more likely than younger adults to choose smaller portions of unhealthy food rather than larger portions of healthy food.

*H4*: The relationship between age and food choice is mediated by consumers’ focal construal (type vs. quantity).

## 3 Study 1

### 3.1 Overview of study 1

The primary goal of Study 1 was to examine whether consumers’ food choices vary depending on their construal level. In Study 1A, we assessed participants’ chronic construal tendencies using the Behavioral Identification Form (BIF) and tested whether these tendencies predicted their food selections. In Study 1B, we experimentally manipulated participants’ construal levels through a priming task to more clearly identify the causal effect of construal level on food choice. Together, these two studies aimed to demonstrate that both chronic and situationally induced construal levels shape consumers’ emphasis on food attributes and ultimately influence their dietary decisions.

### 3.2 Study 1A

#### 3.2.1 Research objective

The objective of Study 1A was to investigate the influence of consumers’ chronic construal level on their food choices. Specifically, we hypothesized that individuals with a high-level construal would be more likely to choose the healthier food option, even if it came with a larger portion, whereas those with a low-level construal would be more inclined to select the less healthy option, even if it was smaller in quantity. This study aimed to explore how consumers’ construal level orientation shapes their decisions in health-related food choice contexts.

#### 3.2.2 Method

##### 3.2.2.1 Participants

Data were collected through an online survey administered via Amazon Mechanical Turk (MTurk). A total of 241 participants initially completed the questionnaire. After excluding 14 responses due to inattentiveness or patterned answering, the final sample consisted of 227 participants. Because this study aimed to examine consumers’ food choice tendencies in the context of health-goal-directed behavior, only adults who could independently set and pursue their own health goals were included. The age of participants ranged from 20 to 61 years (*M* = 37.32, SD = 10.64). Of these, 117 were male (51.5%), 108 were female (47.6%), and 2 did not report their gender.

##### 3.2.2.2 Procedure and stimuli

Participants first completed the Behavioral Identification Form (BIF; Vallacher and Wegner, 1989) to measure their chronic construal level. Each of the 25 items in the BIF presents a common behavior (e.g., “making a list”) and offers two response options: one reflecting a high-level construal (e.g., “organizing things”) and the other a low-level construal (e.g., “writing things down”). Participants selected the option that best described how they perceive the behavior. Responses were coded to calculate an average construal score, ranging from 0 to 1, where higher scores indicated a greater tendency toward high-level construal. Based on these scores, participants were classified into two groups: high-level construal (BIF ≥ 0.5) and low-level construal (BIF < 0.5). After completing the BIF, participants proceeded to a food choice task. They were asked to choose between two snack options: a healthy item with a larger portion (almonds) and an unhealthy item with a smaller portion (chocolate-covered almonds). As shown in Supplementary Figure 1, both options were presented in identical white containers, ensuring that differences in quantity and food type were visually salient and not confounded by packaging or presentation.

#### 3.2.3 Results

To examine whether food choices differed by construal level group (high vs. low), a chi-square test of independence was conducted. The analysis revealed a statistically significant association between construal level and food choice, *χ*^2^(1) = 4.212, *p* < 0.05. Specifically, among participants with a high-level construal, 66.2% (*n* = 102) chose the healthy option with a larger portion (almonds), while 33.8% (*n* = 52) selected the less healthy option with a smaller portion (chocolate-covered almonds). In contrast, participants with a low-level construal showed a more evenly split pattern: 52.1% (*n* = 38) chose the healthy option, and 47.9% (*n* = 35) selected the unhealthy option. These results support the hypothesis that consumers with a higher-level construal are more likely to prioritize food type (healthiness), while those with a lower-level construal tend to focus more on quantity and are relatively more inclined to select the unhealthy but smaller portion alternative. As shown in Table 1, the proportion of participants choosing each food option varied by construal level.

**Table 1:** Food choices by construal level group (Study 1A).

Food Type	Construal level	
	High-level	Low-level
Almonds (Healthy, Large Portion)	102 (66.2%)	38 (52.1%)
Chocolate-covered Almonds (Unhealthy, Small Portion)	52 (33.8%)	35 (47.9%)
Total	154 (100%)	73 (100%)

*χ*^2^= 4.212, *p* < 0.05.

A chi-square test revealed a significant association between construal level and food choice, *χ*^2^(1) = 4.212, *p* < 0.05.

#### 3.2.4 Summary of study 1A

The results of Study 1A indicate that consumers’ construal level tendencies significantly influence food choices. While the overall preference leaned toward the healthier option, participants with a low-level construal were significantly more likely to select the less healthy alternative compared to those with a high-level construal. This suggests that low-construal consumers may attend not only to food type but also to food quantity, leading to a greater likelihood of choosing unhealthy options when portion size becomes salient. These findings highlight construal level as a key psychological mechanism that shapes health-related decision-making in dietary contexts.

### 3.3 Study 1B

#### 3.3.1 Research objective

The primary objective of Study 1B was to extend the findings of Study 1A by testing the causal effect of construal level on food choice through experimental manipulation. Whereas Study 1A examined how individuals’ chronic construal tendencies relate to their food preferences, Study 1B aimed to establish a clearer causal link by directly manipulating construal level. This approach allowed us to assess whether induced differences in construal level would systematically influence participants’ food choices.

#### 3.3.2 Method

##### 3.3.2.1 Participants

Data were collected from a total of 104 participants recruited through Amazon Mechanical Turk (MTurk). Participants ranged in age from 22 to 75 years (*M* = 42.88, SD = 13.84). The sample included 41 males (39.4%), 61 females (58.7%), and 2 participants who did not report their gender.

##### 3.3.2.2 Procedure and stimuli

In Study 1B, participants were primed with either a high-level or low-level construal through situational manipulation, and their subsequent food choices were compared across conditions. Following the methodology of prior studies that have manipulated construal levels through temporal distance (e.g., Trope and Liberman, 2010), participants were asked to imagine a scenario in which they were trying to improve their diet in preparation for an upcoming class reunion. In the high-level construal condition, the reunion was described as taking place 3 months from now, thereby inducing a psychologically distant future and abstract thinking. In the low-level construal condition, the reunion was described as occurring 3 weeks from now, thus creating a sense of temporal proximity and encouraging concrete thinking. After reading the scenario, participants completed a food choice task identical to that used in Study 1A. Specifically, they were asked to choose between a healthy option with a larger portion (almonds) and an unhealthy option with a smaller portion (chocolate-covered almonds). Their choices were then recorded and analyzed based on construal condition.

#### 3.3.3 Results

##### 3.3.3.1 Manipulation check

To verify the effectiveness of the construal level manipulation in Study 1B, participants were asked two items following the priming scenario: (1) “How close in time does the upcoming class reunion feel?” and (2) “How much time do you think is left until the reunion?” Responses to these items were averaged to create a temporal distance perception score. An independent samples t-test revealed a significant difference between conditions. Participants in the high-level construal condition perceived the reunion as significantly farther away (*M* = 3.73, SD = 0.83) than those in the low-level construal condition (*M* = 2.30, SD = 0.83), *t*(102) = 8.729, *p* < 0.001. These results confirm that the manipulation successfully induced the intended psychological distance, thereby validating the construal level manipulation.

##### 3.3.3.2 Hypothesis testing: differences in food choice by construal manipulation

To examine whether food choice varied by construal level condition, a chi-square test of independence was conducted. The analysis revealed a significant difference between the two construal conditions, *χ*^2^(1) = 3.980, *p* < 0.05. Specifically, in the high-level construal condition, 93.8% of participants (*n* = 45) chose the healthy option with a larger portion (almonds), while only 6.3% (*n* = 3) selected the less healthy option with a smaller portion (chocolate-covered almonds). In contrast, in the low-level construal condition, 80.4% of participants (*n* = 45) chose the healthy option, and 19.6% (*n* = 11) opted for the unhealthy alternative. These results support the hypothesis that situationally induced construal levels influence food choice. Participants primed with a high-level construal showed a stronger preference for healthy food options, whereas those in the low-level construal condition were relatively more likely to choose the unhealthy but smaller portion. A summary of food choices by condition is presented in Table 2.

**Table 2:** Food choices by construal level group (Study 1B).

Food type	Construal level	
	High-level	Low-level
Almonds (Healthy, Large Portion)	45 (93.8%)	45 (80.4%)
Chocolate-covered Almonds (Unhealthy, Small Portion)	3 (6.3%)	11 (19.6%)
Total	48 (100%)	46 (100%)

*χ*^2^= 3.980, *p* < 0.05.

Percentages may not total 100 due to rounding. A chi-square test revealed a significant association between construal level and food choice, *χ*^2^(1) = 3.980, *p* < 0.05.

#### 3.3.4 Summary of study 1B

Study 1B investigated whether food choices would differ based on construal level primed through temporal distance manipulation. The results showed that participants in the high-level construal condition tended to focus on the type of food and were more likely to choose the healthy option, even when it came in a larger portion. In contrast, those in the low-level construal condition were relatively more likely to choose the unhealthy option with a smaller portion. These findings suggest that construal level influences which attributes consumers prioritize in health-related decision-making, particularly in terms of attention to food type versus quantity.

## 4 Study 2

### 4.1 Overview of study 2

The primary objective of Study 2 was to examine whether construal level varies by age and whether age-related differences influence attentional focus and food choice. Specifically, this study aimed to investigate whether individuals’ food selections—between a healthy option with a larger portion and an unhealthy option with a smaller portion—differ depending on age. Furthermore, it sought to explore how attentional focus on food quantity versus type mediates the relationship between age and dietary decisions.

### 4.2 Study 2A

#### 4.2.1 Research objective

The objective of Study 2A was to examine the effect of age on consumers’ construal level. Prior research suggests that as individuals age, they tend to focus more on concrete and practical information, while preferring simple and intuitive content over complex or abstract information (Mather and Carstensen, 2005; Park, 2002; Steers, 2014). Building on these findings, the present study investigated whether older adults are more likely to exhibit a low-level construal tendency compared to younger individuals.

#### 4.2.2 Method

##### 4.2.2.1 Participants

Data for Study 2A were collected from a total of 105 participants recruited via Amazon Mechanical Turk (MTurk). Participants ranged in age from 20 to 61 years (*M* = 37.46, SD = 9.87). The sample consisted of 51 males (48.6%), 53 females (50.5%), and 1 participant who did not report their gender.

##### 4.2.2.2 Procedure and stimuli

Participants’ construal level tendencies were measured using the Behavioral Identification Form (BIF), following the same procedure as in Study 1A. The BIF consists of 25 items, each presenting a common behavior (e.g., “making a list”) accompanied by two response options—one reflecting a high-level construal and the other a low-level construal. Participants were instructed to select the option that best represented how they typically interpret each behavior. Responses were recoded and averaged to produce a mean BIF score for each participant, ranging from 0 to 1. Higher scores indicated a stronger tendency toward high-level construal. Based on these scores, participants were categorized into either a high-level or low-level construal group. Age was also measured and subsequently used as a predictor variable in the analysis.

#### 4.2.3 Results

To examine whether age significantly predicted construal level, a binary logistic regression analysis was conducted. Age was entered as the independent variable, and construal level served as the dependent variable (0 = low-level construal, 1 = high-level construal). The analysis tested whether an increase in age would be associated with a decreased likelihood of exhibiting a high-level construal.

Results revealed a significant effect of age on construal level. Specifically, age negatively predicted the likelihood of high-level construal (*B* = −0.044, SE = 0.022, Wald = 4.045, *p* < 0.05), indicating that older participants were more likely to adopt a low-level construal. A summary of the regression results is presented in Table 3.

**Table 3:** Logistic regression results predicting construal level from age.

	B	SE	Wald	df	*p*	Exp(B)
Age	−0.044	0.022	4.045	1	0.044*	0.0957
Constant	2.407	0.870	7.645	1	0.006**	11.097

*p < 0.05, **p < 0.01.

Dependent variable: construal level (0 = low-level, 1 = high-level).

#### 4.2.4 Summary of study 2A

Study 2A examined the relationship between age and consumers’ construal level. The findings indicated that as age increases, individuals are less likely to adopt an abstract, high-level construal and more likely to rely on a concrete, low-level construal. These results suggest that older adults may prioritize practical, actionable information over complex or long-term health-related concepts, highlighting a shift in cognitive processing tendencies with age.

### 4.3 Study 2B

#### 4.3.1 Research objective

The primary objective of Study 2B was to investigate whether consumers’ attentional focus and food choices differ by age. Specifically, this study examined whether individuals of different ages show systematic differences in their food selections—between a healthy option with a larger portion and an unhealthy option with a smaller portion—and whether attentional focus on food quantity versus food type mediates this relationship.

#### 4.3.2 Method

##### 4.3.2.1 Participants

Data for Study 2B were collected through a nationally representative online panel managed by Embrain, a leading research agency in South Korea. The final sample consisted of 300 participants, with an equal gender distribution: 150 males (50%) and 150 females (50%). Participants ranged in age from 20 to 68 years (*M* = 44.15), and the age distribution was relatively balanced across the sample.

##### 4.3.2.2 Procedure and stimuli

Participants were first given a brief introduction regarding the importance of health management. They were then presented with a food choice task in which they were asked to select between two snack options: a larger portion of pretzels (healthy option) and a smaller portion of chocolate-covered pretzels (less healthy option). These two options were designed to differ in perceived healthiness and quantity, while maintaining similar calorie content. The stimuli used in this task are shown in Supplementary Figure 2. Following the food choice task, participants responded to two items assessing their attentional focus during decision-making. Using a 7-point Likert scale, they were asked: (1) “How important was the type of food in your decision?” (2) “How important was the quantity of food in your decision?” Participants also completed measures of age and health involvement, which were used as covariates in the subsequent analysis.

#### 4.3.3 Results

To examine whether attentional focus on food quantity and type mediates the effect of age on food choice, a mediation analysis was conducted controlling for participants’ level of health involvement. The dependent variable was food choice (1 = healthy option with a larger portion [pretzels], 2 = less healthy option with a smaller portion [chocolate-covered pretzels]). The overall model was statistically significant. Age had a significant direct effect on food choice (*B* = 0.048, SE = 0.010, *Z* = 4.59, *p* < 0.001), indicating that as age increased, participants were more likely to choose the less healthy option (chocolate-covered pretzels). Among the two mediators, focus on quantity had a significant positive effect on choosing the less healthy option (*B* = 0.265, SE = 0.116, *Z* = 2.28, *p* = 0.022), whereas focus on type had a significant negative effect (*B* = −0.347, SE = 0.127, *Z* = −2.74, *p* = 0.006).

**Figure 1:**
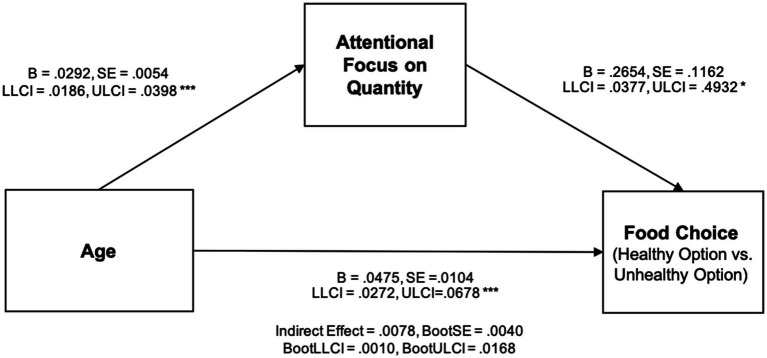
Mediation model: the effect of age on food choice through attentional focus on quantity.

Moreover, the indirect effect of age on food choice through focus on quantity was statistically significant (indirect effect = 0.0078, 95% CI: [0.0010, 0.0168]). In contrast, the indirect effect through focus on type was not significant. These findings suggest that older adults tend to focus more on the quantity of food rather than its type when making dietary decisions. This shift in attentional focus increases their likelihood of selecting the less healthy but smaller option. In other words, the impact of age on food choice is significantly mediated by attentional focus on quantity, reflecting an age-related shift toward more concrete, quantity-based evaluation criteria. The full mediation model and path coefficients are illustrated in Figure 1.

#### 4.3.4 Summary of study 2B

Study 2B examined whether attentional focus tendency mediates the relationship between age and food choice. The results showed that as age increased, individuals were significantly more likely to choose the less healthy food option (chocolate-covered pretzels). Furthermore, older participants tended to place greater importance on food quantity when making dietary decisions. This quantity-focused tendency significantly mediated the relationship between age and the likelihood of choosing the less healthy option. These findings suggest that with increasing age, consumers are more likely to evaluate food based on concrete and measurable attributes—such as quantity—rather than abstract attributes like food type. This shift in attentional focus highlights the importance of considering age-related cognitive processing styles in understanding food choice behavior.

## 5 Discussion

### 5.1 Conclusion

This study aimed to examine how consumers’ construal levels influence their focus on food attributes—specifically food type and quantity—and how these attentional patterns subsequently shape food choices for health management. Moreover, the study explored how these effects vary across age groups, based on the premise that construal level shifts with age.

Health-related food choices are often determined by two primary attributes: food “type” (e.g., healthy vs. unhealthy) and food “quantity” (e.g., large vs. small portions). Building on construal level theory, we hypothesized that the degree to which consumers focus on these attributes depends on their construal level. High-level construals are associated with abstract, goal-related thinking and therefore a stronger emphasis on food type (virtue vs. vice), whereas low-level construals are linked to concrete, detail-oriented thinking and thus a greater focus on food quantity.

Across two experimental studies, the findings revealed two central outcomes. First, consistent with prior research, food type information generally exerted a dominant influence on choice across conditions. Participants were more likely to choose larger portions of healthy foods. However, this pattern was moderated by construal level: individuals primed with or chronically holding a low-level construal exhibited increased attention to quantity, resulting in more frequent selection of smaller, yet less healthy options. This demonstrates that attribute focus can shift depending on the consumer’s construal mindset, thereby altering their food decisions.

Second, we found that age plays a significant role in shaping food attribute focus. Older adults were more likely to adopt a lower-level construal, leading them to prioritize food quantity over type. As a result, they showed a higher tendency to choose smaller portions, even when these options were unhealthy. While the predicted increase in type-based focus among younger adults was not statistically robust, the results overall support the notion that age-related differences in construal level contribute to distinct patterns of food choice.

Together, these findings suggest that construal level is a key psychological mechanism driving food decision-making and that age-related changes in construal level may partly explain the variability in health-related choices. These insights provide meaningful implications for designing age-sensitive interventions and health communication strategies aimed at promoting better dietary decisions across the lifespan.

### 5.2 Theoretical and managerial implications

This study provides both theoretical and practical implications for understanding food choice in health-related contexts. First, it elucidates the cognitive mechanism through which consumers’ focus on specific food attributes—namely, food type versus quantity—affects actual choice behavior. Drawing on construal level theory (CLT), the study demonstrates that consumers at a higher construal level are more likely to focus on food type (a categorical, abstract attribute), whereas those at a lower construal level attend more to food quantity (a concrete attribute). Furthermore, we show that age systematically influences this focus, as older adults tend to adopt lower-level construal, leading to greater attention to quantity. These findings contribute to the literature by clarifying how construal level mediates the relationship between age and food attribute preference, thereby identifying a core psychological mechanism underlying age-related differences in food decision-making.

Second, the study confirms that food type is prioritized over quantity when individuals are operating under a health management goal. This suggests that in health-relevant contexts, abstract or categorical attributes tend to dominate concrete ones in guiding consumer attention and judgment. The results imply that activating a health-related goal naturally induces a high-level construal, prompting consumers to focus on food type. This supports the broader proposition that goal salience shifts individuals into a higher construal mindset. Accordingly, persuasive health messages may be more effective when their content aligns with the activated construal level—that is, when there is a fit between the message framing and the consumer’s mental representation level.

Third, by demonstrating that age-related variations in food choice can be explained by differences in construal level, this research offers actionable insights for designing age-tailored persuasive strategies in health communication. Specifically, for older adults, health messages should highlight concrete, actionable, and easy-to-implement behaviors based on lower-level construal. In contrast, for younger individuals, messages emphasizing abstract values, long-term benefits, and ideal health goals—consistent with high-level construal—may be more persuasive. These insights provide a conceptual foundation for developing customized health communication strategies based on the construal tendencies associated with different age groups.

### 5.3 Limitations and future research

Despite the theoretical and practical implications of this study in the context of health communication, several limitations must be acknowledged, along with suggestions for future research. First, although this study empirically validated the psychological mechanism of construal level through the focus on food attributes, it did not directly measure age-related differences in construal level. While previous literature suggests that age differences in attentional focus may reflect underlying construal level differences, future studies should incorporate direct measures of construal level (e.g., the BIF) to strengthen the explanatory power regarding the focus on food type versus quantity.

Second, while this study confirmed that older adults’ food choices are more influenced by quantity-focused attributes, it did not find strong evidence that younger adults’ decisions were predominantly guided by food type. This may be because, in the context of health-related decision-making, the distinction between healthy and unhealthy food types (i.e., labeling) serves as universally salient information regardless of age (Woolley and Liu, 2021). Therefore, the lack of a clear age-based difference in food type focus may reflect that all participants prioritized this attribute. Furthermore, this finding aligns with Hadar et al. (2021), who noted that older adults are more likely to incorporate secondary cues in decision-making processes. It is possible that while both age groups processed food type similarly, older adults were more likely to additionally consider lower-level attributes such as quantity. However, this makes it difficult to conclude that younger adults uniquely or more strongly emphasized food type. Future research should consider experimental manipulations and measures that more clearly distinguish the relative importance of type versus quantity across age groups.

Third, the manipulation of temporal distance in Study 1B, conducted under controlled laboratory conditions, may not fully reflect real-world decision-making contexts. Participants were asked to imagine a reunion after a given time interval, which might not fully represent the complex situational factors that naturally influence construal-level shifts in real life. Although this procedure followed established CLT paradigms, future studies should enhance real-world validity and better capture natural variations in construal level. In addition, the study aimed to understand how people select foods based on attribute focus in everyday health-related decisions. For this purpose, specific food stimuli (almonds and pretzels) were chosen based on prior studies. However, the use of snack-type foods presents a limitation in generalizability. Future research should explore a broader range of food categories to enhance external validity. Moreover, this study examined food choice at a single-item level, without considering how individual selections fit within a broader dietary context or habitual eating patterns. Future research should investigate how construal level and age jointly influence overall meal composition and daily dietary balance. In addition, future studies should assess the extent to which participants perceive these foods as aligned with their personal health goals.

Fourth, although chocolate-based foods were used to represent “unhealthy” options, this classification was limited to a single food type. While chocolate is commonly treated as an unhealthy product in prior studies, this narrow operationalization requires further scrutiny. It remains unclear how strongly chocolate is perceived as unhealthy across diverse consumers and whether other “unhealthy” food cues (e.g., high-fat, high-sugar, fried foods) might evoke different responses. Future studies should test a broader range of food stimuli with varying negative attributes to enhance generalizability. Furthermore, the operational definition of “unhealthy” food in this study was largely calorie-based and may not fully capture culturally or nutritionally diverse perceptions of food healthiness. Additionally, this study employed food stimuli and health-based categorizations grounded in prior international research. Future studies should examine how cultural differences in dietary norms and health perceptions influence the construal-based mechanisms of food choice.

Fifth, in Study 2B, the focus of attention on food type and quantity was assessed through self-report. However, self-reported measures may not fully capture the implicit cognitive or affective processes that drive actual food decisions, as individuals may not always be consciously aware of their attentional focus. Future research should employ complementary methods such as eye-tracking, reaction-time tasks, or behavioral indicators to more objectively assess attentional mechanisms in food-choice contexts.

Sixth, this study focused on a one-time food choice. However, future research should move beyond single-item selections and consider how food choices are composed in larger dietary contexts. Further investigation into post-choice behaviors (e.g., licensing effects) could also provide insights into whether the selected food contributes meaningfully to health goal attainment. Moreover, researchers should examine how consumers respond to different food attributes such as ingredients (reflecting high-level construals) and cooking methods (reflecting low-level construals). Such inquiry would offer deeper insights into how food-related decisions are guided by construal level in everyday health management.

Lastly, although this study focused on food choices, health behaviors encompass a wide array of activities such as exercise. Exercise choices, too, can be framed in terms of type (e.g., strength training vs. cardio) and quantity (e.g., duration). Investigating whether the effects of construal level and age observed in food choice generalize to exercise behavior would contribute to a more comprehensive understanding of age-related differences in health decision-making.

## Data Availability

The raw data supporting the conclusions of this article will be made available by the authors, without undue reservation.
