# Supplementary material for: “Same Calories, Different Foods” – What do you choose? The role of construal level and age in shaping food choices
Source: Front Psychol. 2025 Oct 29;16:1688277. doi: 10.3389/fpsyg.2025.1688277 (PMC12605454; doi:10.3389/fpsyg.2025.1688277)
Supplement: Supplementary file 1 [file Supplementary_file_1.docx]

Supplementary Material

**Supplementary Figure 1.** Example of food choice task stimuli (almonds vs. chocolate-covered almonds).


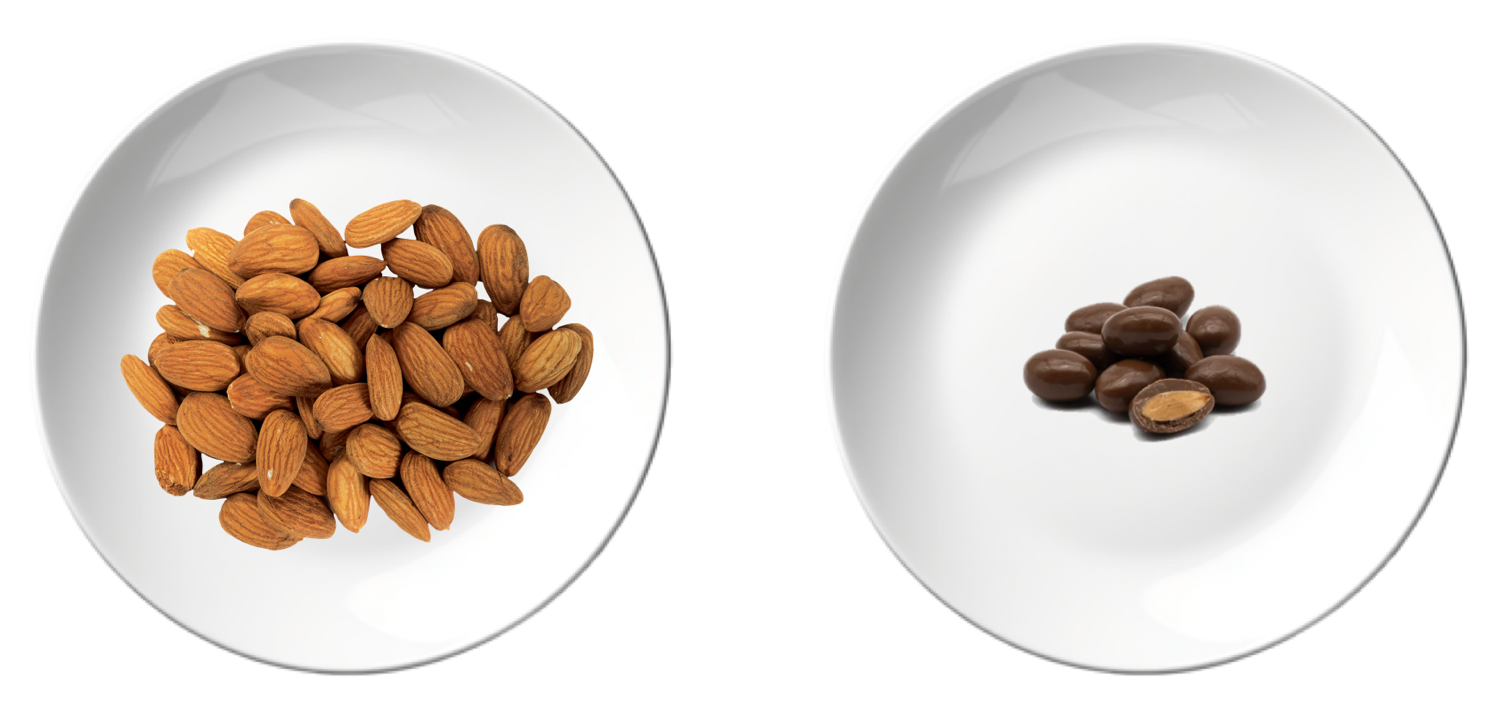


Both options were presented in identical white containers to isolate differences in quantity and food type from packaging effects.

*Source: Adapted and restructured by the authors based on Woolley, K., & Liu, P. J. (2021). “How You Estimate Calories Matters: Calorie Estimation Reversals.” Journal of Consumer Research, 48(1), 147–168. https://doi.org/10.1093/jcr/ucaa059*

**Supplementary Figure 2.** Example of food choice task stimuli (pretzels vs. chocolate-covered pretzels).


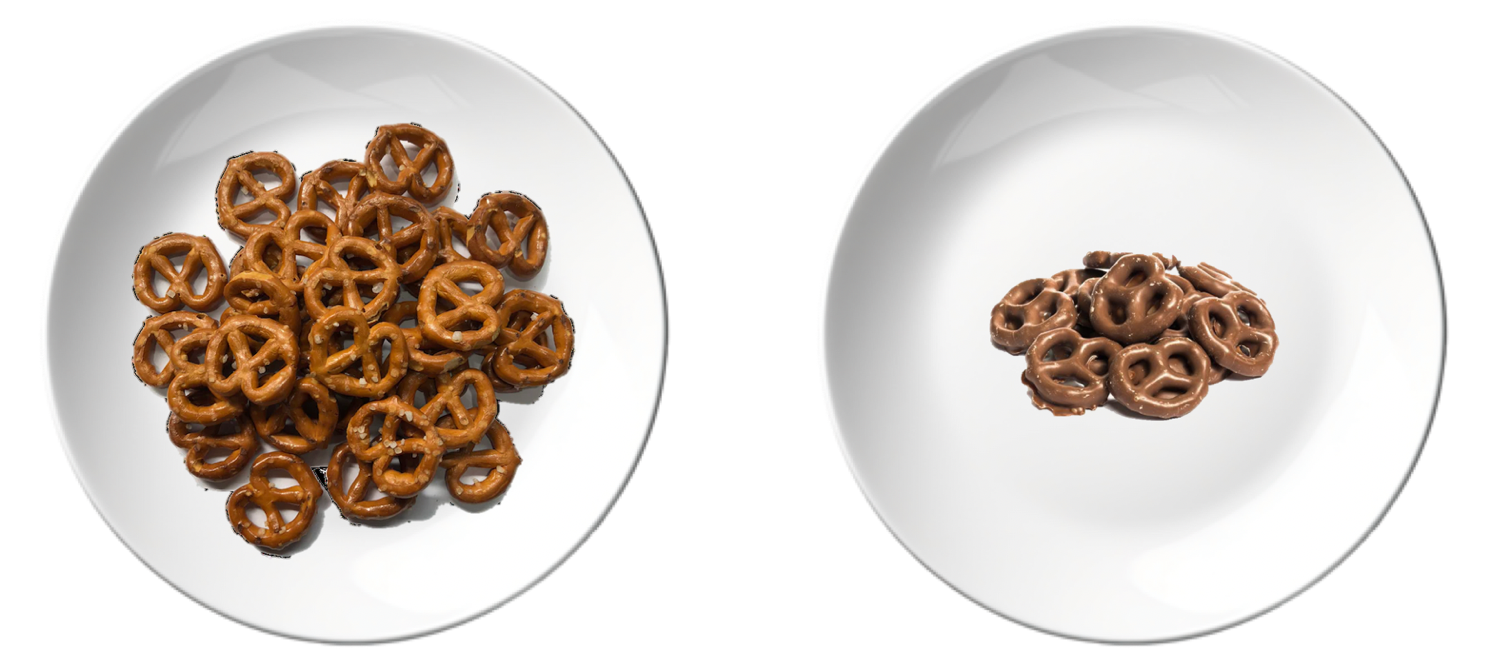


Both options were presented in identical white containers to ensure that visual differences were attributable only to quantity and food type.

*Source: Adapted and restructured by the authors based on Woolley, K., & Liu, P. J. (2021). “How You Estimate Calories Matters: Calorie Estimation Reversals.” Journal of Consumer Research, 48(1), 147–168. https://doi.org/10.1093/jcr/ucaa059*
